# Curettage for Copious Conjunctival Concretions

**DOI:** 10.7759/cureus.11742

**Published:** 2020-11-28

**Authors:** Danny Lam, Elizabeth L Wong, Ashish Agar, Minas T Coroneo, Ian C Francis

**Affiliations:** 1 Ophthalmology, Sydney Hospital and Sydney Eye Hospital, Sydney, AUS; 2 Ophthalmology, Prince of Wales Hospital, Sydney, AUS

**Keywords:** curettage, concretions, oculoplastics

## Abstract

Management of multiple exposed eyelid concretions can be performed successfully in an anesthetized eyelid with gentle curettage of the concretions using a small chalazion curette. It has the advantage of managing the patient supine, providing better eyelid stability and visibility, and minimizing risk in the event of patient movement. This curettage technique was used to facilitate the successful resolution of a patient’s ocular surface irritative symptomatology due to multiple exposed concretions.

## Introduction

Conjunctival concretions are small yellow-white nodules usually located on the palpebral conjunctiva of the upper and lower eyelids [1]. They are quite common, as shown by a study from an English Eye Emergency department demonstrating a prevalence of 42% in 100 consecutive patients [2]. Concretions are typically inactive and simply found in elderly patients or patients with chronic conjunctival inflammation; however, they can also occur in younger individuals with no significant ophthalmological disorders [3]. There is also an association between conjunctival concretions and Meibomian gland dysfunction [4].

Conjunctival concretions are usually asymptomatic. These concretions are often only noticed incidentally unless the concretions erode the conjunctiva and become symptomatic by causing a corneal abrasion. These symptoms include foreign body sensation, tearing, redness, photophobia, and blurred vision [1]. Exposure of concretions occurs in 7% of patients [2].

Microscopy shows that concretions are composed of degenerated epithelial cells and mucinous secretions, with strong staining for phospholipid and elastin and weak staining for polysaccharides and lipids [5]. The pathogenesis is considered to relate to normal aging or to be associated with eyelid inflammation, such as occurs in trachoma, various types of keratoconjunctivitis, or dry eye disease, including the Meibomian gland dysfunction. The degenerated epithelial cells with their associated debris, and inspissated proteinaceous secretions such as keratin, are deposited in the subepithelial space or conjunctival recesses of Henle’s glands [1]. Perhaps surprisingly, research has not confirmed the presence of either calcium or phosphate [5].

Macroscopic examination with eversion of the upper and lower eyelids allows concretions to be recognized easily. Their characteristics are confirmed under biomicroscopy in the clinic. Fluorescein staining permits concretions that have eroded through the palpebral conjunctiva to be easily visualized with cobalt blue light. The exposed concretions protruding through the conjunctiva are thought to produce the symptoms described above.

Most concretions remain asymptomatic and do not require specific intervention. If the patient is symptomatic and has just a few exposed concretions, these can be removed with topical anesthesia (such as oxybuprocaine hydrochloride 0.4%) and a 25G sterile needle at the biomicroscope [1]. The authors also ensure that the patient’s head is totally stabilized by a nurse or orthoptist during this procedure, minimizing the risk of a penetrating eye injury with the needle.

When the patient has dozens of concretions, it is impractical to remove them one-by-one on the biomicroscope. As an alternative, the authors have used a straightforward curettage technique, which has produced excellent results without the difficulties of patient posturing and stabilization sitting upright on the biomicroscope. Curettage of the eyelid using a chalazion curette is an established, gentle, and effective technique commonly used as part of the management of chalazia and in the treatment of lacrimal canaliculitis [6, 7].

This report demonstrates the ready applicability of gentle conjunctival curettage after appropriate topical and eyelid infiltration anesthesia for the removal of multiple exposed concretions in patients’ upper and lower eyelids [8]. It appears to facilitate a satisfactory clinical outcome.

This work was conducted in accordance with the National Statement on Ethical Conduct in Human Research and is consistent with the principles that have their origin in the Declaration of Helsinki. Written signed consent was obtained to reveal protected health information and to use photographs of the patient.

## Case presentation

A 70-year-old Middle Eastern male presented for ophthalmological diagnosis. He described the sensation in both eyes as ‘pins and needles’, which had been present for at least four months. Despite the use of preservative-free lubricating eye drops and ointment, his symptomatology had not improved.

Systemically, the patient had well-controlled non-insulin-dependent diabetes mellitus and obstructive sleep apnoea but was otherwise generally well. His only ophthalmic history was that of successful bilateral cataract and intraocular lens implantation surgery three years prior to this presentation.

His best-corrected visual acuity was 6/6 in both eyes with intraocular pressures of 15 mmHg in the right eye and 16 mmHg in the left eye. Considering the patient’s sensory symptomatology, a dedicated and thorough neuro-ophthalmological examination revealed no evidence of a structural lesion, which could have caused paraesthesiae of the patient’s eyes or eyelids.

**Figure 1:**
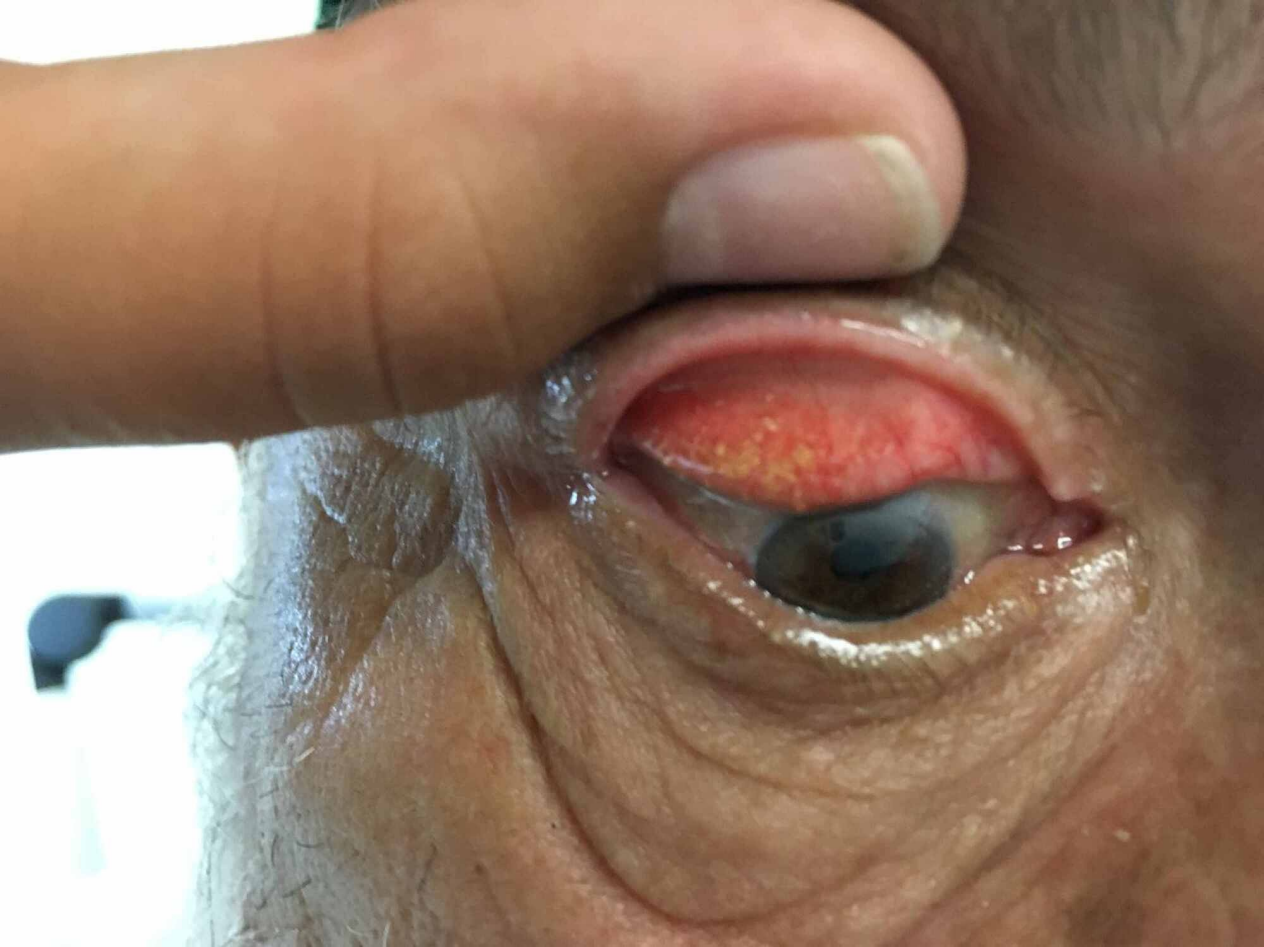
Multiple concretions on right upper eyelid eversion

**Figure 2:**
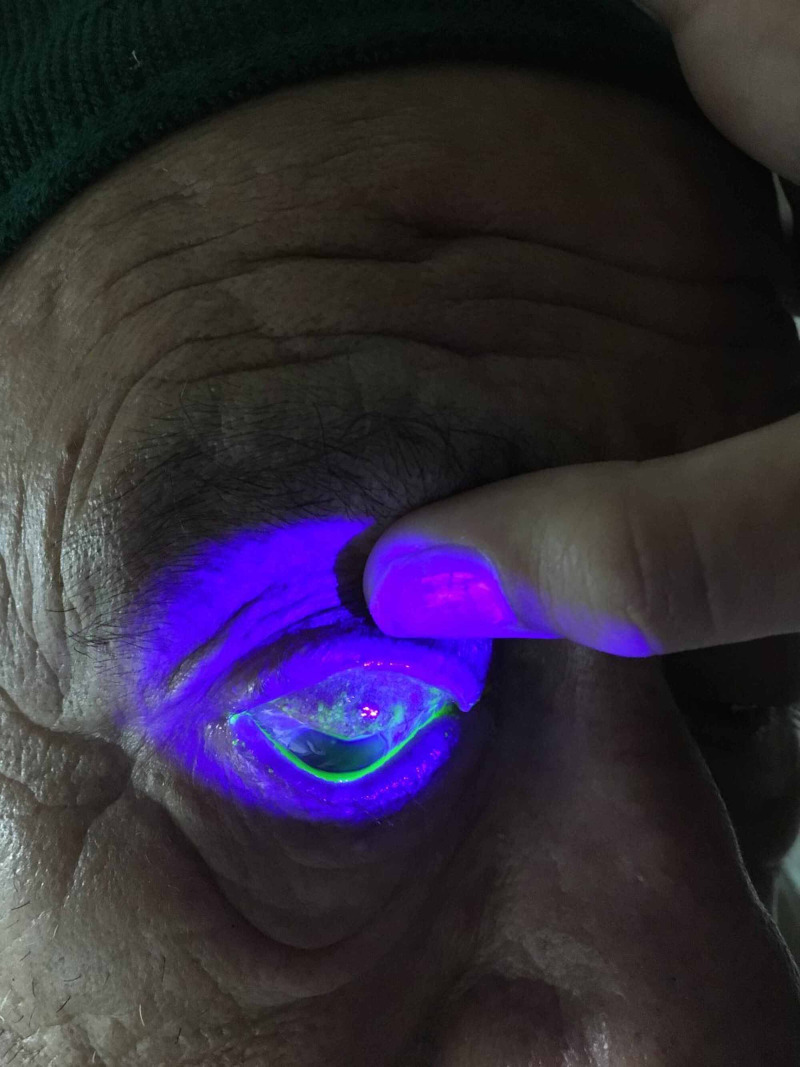
Fluorescein staining of multiple exposed concretions on the right upper eyelid

As no corneal foreign bodies were seen, an everted eyelid examination was carried out. This demonstrated approximately 50 concretions on the right upper eyelid (Figure 1), 20 on the left upper eyelid, as well as numerous bilateral lower eyelid conjunctival concretions. While there was no evidence of corneal ulceration on fluorescein staining, there was substantial fluorescein staining of nearly all of the upper and lower eyelid exposed concretions (Figure 2).

 

Given that the exposed concretions were symptomatic, the decision was made to remove them. Operating loupes were used to facilitate visualization during the procedure. With the patient supine and following topical anesthesia of the corneal surface with oxybuprocaine hydrochloride 0.4%, the patient underwent infiltration anesthesia of all four eyelids via a trans-conjunctival approach, using the Facilitated Lid Anaesthesia Technique (FLAT) [8], and 2% lignocaine with 1/80,000 adrenaline. A chalazion clamp was used to evert and stabilize the eyelids sequentially. A small chalazion curette was employed using a gentle curettage technique, easily removing the numerous visible and exposed concretions from upper and lower eyelids. Hemostasis was achieved with gentle pressure over the tarsoconjunctiva. The patient was asked to use topical antibiotic eye drops for one week with a review planned at six weeks.

At the review, the patient’s ‘pins and needles’ sensation had totally resolved. There were two residual buried concretions in the right upper eyelid and four in the left upper eyelid; however, none of these appeared likely to cause any ocular surface symptomatology due to their locations and low likelihood for potential exposure. There was no scarring, impairment of conjunctival healing, or any other potential complications.

The patient was discharged and asked to use only preservative-free lubricating eye drops. He was informed that while he did have several residual concretions, these did not currently need to be removed. He was also advised that if he developed any further ocular surface symptoms related to exposure of these concretions, he could most likely have them managed with the standard needle technique using topical anesthesia on the biomicroscope.

## Discussion

This report demonstrates a simple and effective method of removing multiple exposed eyelid concretions, seen with fluorescein staining and cobalt blue light, using gentle curettage with a small chalazion curette. While the use of topical anesthesia with the needle technique is recommended for the removal of small numbers of exposed concretions using the biomicroscope, it is not feasible for patients with multiple exposed concretions.

Firstly, topical anesthesia may not provide sufficient analgesia for the removal of copious numbers of concretions. Secondly, as upper eyelid concretions are removed, the conjunctiva may bleed, reducing the visibility of nearby concretions, and especially those inferior to the concretions being removed. Not only does this make their needle removal more difficult, but the blood has to be continually removed to facilitate visualization of the remaining concretions. In practice, this is usually done by the assistant stabilizing the patient’s head and using a cotton bud or surgical spear to remove the blood from the surgical field. This can make the process quite time-consuming and potentially uncomfortable for the patient.

This gentle curettage technique provides effective, safe and rapid removal of multiple conjunctival concretions. It has the advantage of managing the patient supine during the topical and eyelid infiltration anesthesia. It also minimizes the risk of patient movement that could occur during individual concretion removal with a needle on the biomicroscope, as each head movement has the possibility of exposing the patient to globe trauma or perforation by the needle. Further, the surgical field is much better visualized and is much more stable, as the patient is supine and not vertical on the biomicroscope. Standard hemostatic techniques such as the use of cotton buds or sterile gauze may be used to maintain constant visibility of the concretions.

The FLAT technique [8] of lid infiltration anesthesia of the eyelid not only minimizes pain during the curettage procedure but also optimizes hemostasis. Moreover, the patient is not compelled to keep the chin and forehead against the biomicroscope chinrest and forehead bar. Thus, not only is patient head movement avoided, but the possibility of a vasovagal event is minimized.

## Conclusions

This technique allows safe and efficient removal of copious numbers of exposed concretions from the eyelids, a procedure that would be tedious and time-consuming when performed at the slit lamp.
